# Necrotizing Fasciitis

**Published:** 2014-05-17

**Authors:** Sameer Gujral, Juliana Maria Fonseca Hughes, Akira Wiberg

**Affiliations:** Department of Plastic Surgery, Derriford Hospital, Plymouth, United Kingdom

**Keywords:** cellulitis, necrotizing fasciitis, myositis, sepsis, *Streptococcus*

## DESCRIPTION

A 28-year-old woman with a raised body mass index presented feeling unwell with a 16-hour history of increasing pain of her left medial thigh. Her white cell count, C-reactive protein (CRP) and lactate levels were elevated. A diagnosis of necrotizing fasciitis was made, and the patient was promptly taken to the operating theatre.

## QUESTIONS

1. **What findings differentiate a severe cellulitis from necrotizing fasciitis?**
2. **What are the common causative organisms and what antibiotic therapy is indicated?**
3. **What risk factors predispose individuals to this condition?**
4. **What treatment is indicated?**

## DISCUSSION

Necrotizing fasciitis is a severe, rapidly spreading, and potentially life-threatening infection of soft tissues. The infection spreads along fascial planes and may spare the skin and the muscle. Patients present with a history of rapid onset of localized pain and redness or discolored skin, with or without a precipitating breach of the skin. Unlike patients with cellulitis, patients frequently present in extremis, with evidence of septic shock or early signs of multiorgan dysfunction. Common findings on examination include rapidly advancing erythema, which may be non-blanching, soft-tissue swelling, severe pain, hemorrhagic blistering, and palpable crepitus. In laboratory tests, white cell count and CRP levels are usually greatly elevated in necrotizing fasciitis and there may also be hyponatremia, lactic acidosis, and coagulopathy. LRINEC (Laboratory Risk Indicator for Necrotizing Fasciitis) is a scoring system that aims to differentiate between cellulitis and necrotizing fasciitis. It is based on 6 routinely measured markers (white cell count, CRP, sodium, hemoglobin, creatinine, and glucose), and a score of 6 or greater has a positive predictive value of 92.0% and a negative predictive value of 96.0% for diagnosing necrotizing fasciitis.^1^

Necrotizing fasciitis is usually polymicrobial in nature. When caused by a single agent, this is usually group A β-hemolytic streptococci. The other microorganisms implicated are anaerobes (such as clostridia) and gram-negative rods.^2^ As these can be gas-forming, surgical emphysema may be visible on radiographs or palpable on examination. Prompt broad-spectrum antibiotic therapy is crucial and must be effective against gram-positive and negative organisms as well as anaerobes. Clindamycin is the most effective agent against group A streptococci, which may be responsible for streptococcal toxic shock syndrome^3^; it is therefore frequently used in conjunction with broad-spectrum antibiotics (such as carbapenems) in accordance with institutional therapeutic protocols. Following microbiological analysis of tissue samples, antibiotic therapy should be targeted toward the causative organisms.

Risk factors that are known to be associated with necrotizing fasciitis include immunocompromised state (including long-term steroid use), diabetes mellitus, peripheral vascular disease, obesity, intravenous drug abuse, smoking, and extremes of age. However, the condition can also affect young, healthy patients either unexpectedly or following a routine surgical procedure.

Surgery is the mainstay of urgent and life-saving treatment. Excision of the involved soft tissues down to healthy, bleeding, and uninfected margins is imperative. In the operating theatre, exploratory incisions are made to assess the extent of the disease. Common intraoperative findings include a gray “dishwater” fluid in the fascial plane and necrotic, thrombosed subcutaneous fat that can easily be swept off the fascia. Patients frequently require multiple returns to the operating theatre for further debridement; the resulting defects are reconstructed once the infection has resolved. In the case illustrated, the defect on the thigh was resurfaced with a split-thickness skin graft. Adjunctive treatments that may be advocated in necrotizing fasciitis include immunoglobulins^4^ in the context of streptococcal toxic shock syndrome and hyperbaric oxygen therapy,^5^ where available and appropriate.

Necrotizing fasciitis has a high mortality rate, estimated at 25% to 35%.^6^ Optimal outcome relies on early diagnosis and expedient surgical debridement, coupled with targeted intravenous antibiotic therapy and intensive care support.

## Figures and Tables

**Figure 1:**
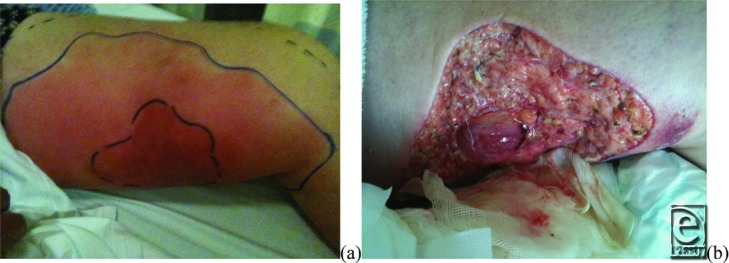
(a) Left medial thigh showing discoloration and swelling. Maximal pain to palpation was in the centrally marked area, but pain could be elicited as far back as the dotted lines. (b) Left medial thigh following surgical debridement down to viable fat, fascia, and muscle.
